# The bridge helix coordinates movements of modules in RNA polymerase

**DOI:** 10.1186/1741-7007-8-141

**Published:** 2010-11-29

**Authors:** Pyae P Hein, Robert Landick

**Affiliations:** 1 Department of Biochemistry, University of Wisconsin-Madison, 5441 Microbial Sciences, 1550 Linden Drive, Madison, WI 53706, USA; 2 Department of Bacteriology, University of Wisconsin-Madison, 5441 Microbial Sciences, 1550 Linden Drive, Madison, WI 53706, USA

## Abstract

The RNA polymerase 'bridge helix' is a metastable α-helix that spans the leading edge of the enzyme active-site cleft. A new study published in *BMC Biology *reveals surprising tolerance to helix-disrupting changes in a region previously thought crucial for translocation, and suggests roles for two hinge-like segments of the bridge helix in coordinating modules that move during the nucleotide-addition cycle.

See Research article: http://www.biomedcentral.com/1741-7007/8/134

## 

DNA-dependent, multisubunit RNA polymerases are conserved in core structure and are required for gene expression and regulation in all cellular organisms. To accomplish these roles, RNA polymerase has evolved into a complex molecular machine in which precisely orchestrated movements in a network of flexible modules mediate steps in a nucleotide-addition cycle of four basic steps: translocation of RNA and DNA chains through the polymerase; binding of the nucleotide triphosphate (NTP) substrate; catalysis; and pyrophosphate release. A key component of this network is the bridge helix, which occupies a critical position spanning the main channel of RNA polymerase just downstream of the active site (Figure 1a). After the first crystal structures of RNA polymerases emerged, the bridge helix garnered immediate attention as a possible effector of translocation, both because of its central location and because in RNA polymerases that are not bound to DNA, it partially unfolds to form a loop that clashes with the position of the templating DNA base in DNA-containing RNA polymerase structures (Figure 1b) [1]. Formation of the bridge helix loop is proposed to act as the pawl in a ratchet-like translocation mechanism to move DNA through RNA polymerase [2]. Subsequent crystal structures of NTP-bound elongation complexes suggested that the bridge helix might also play a role in catalysis [3-5]. In such structures, a continuous bridge helix forms a three-helix bundle with a neighboring domain known as the trigger loop, which folds into the trigger helices that contact NTP substrate in an NTP-bound elongation complex. Because positioning of the NTP substrate by trigger-helix contacts is required for efficient catalysis, even small movements of the bridge helix, not necessarily involving unfolding, may modulate catalysis by favoring or disfavoring formation of the trigger helices [3,5].

**Figure 1:**
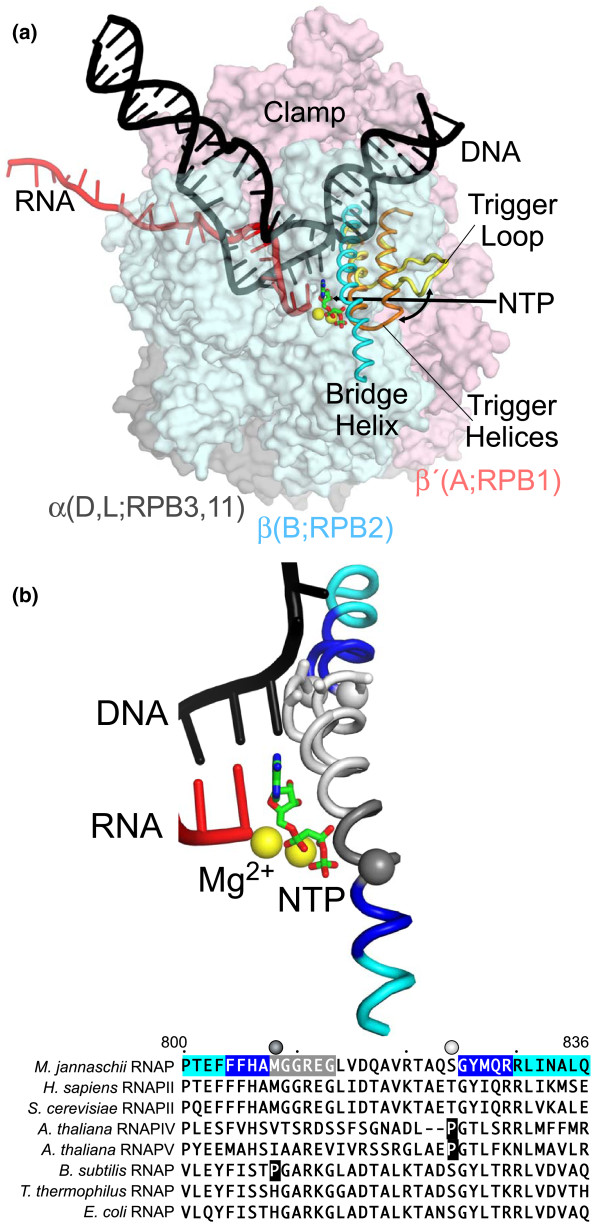
**Positions and conformations of the bridge helix in an elongation complex**. **(a) **Structure of an elongation complex based on the crystal structure of a NTP-bound RNA polymerase from *Thermus thermophilus *(PDB 2o5j) [3]. DNA (black) is melting into a transcription bubble that allows template-strand pairing with RNA (red) in a 9-10 base pair RNA-DNA hybrid. The bridge helix (cyan) and trigger loop/helices (yellow/orange) lie on the downstream side of the active site. The presumed path of NTP entry is indicated by the straight arrow. Interconversion of the trigger loop and trigger helices is indicated by the curved arrow. The RNA polymerase subunits are shown as semi-transparent surfaces with the identities of orthologous subunits in bacteria (α, β, and β', gray, blue, and pink, respectively), archaea (D, L, B, and A), and eukaryotic RNA polymerase II (RPB3, 11, RPB2, RPB1) indicated. The active site Mg^2+ ^ions are shown as yellow spheres, and α,β-methylene-ATP in green and red. **(b) **Conformations of the bridge helix observed on crystal structures of a NTP-bound elongation complex and of an RNA polymerase lacking nucleic acids. The positions of nascent RNA, the template DNA strand, α,β-methylene-ATP, Mg^2+^, and straight bridge helix are from the PDB 2o5j structure. The looped-out bridge helix indicating the conformation in a nucleic-acid-free structure is from *T. thermophilus *RNA polymerase bound by σ^A ^initiation factor (PDB 1iw7). Positions at which substitutions with proline increase polymerase activity are marked by Cα spheres (H_N _and H_C_) [7]. The location of a deletion of two amino acids in the plant RNA polymerase IV enzyme is marked by Cα-Cβ sticks (next to the white sphere marking the proline substitution). Sequences of the bridge helix from several RNA polymerases are shown, with the *M. jannaschii *bridge helix color-coded as in the molecular model: blue, segments in which two-amino-acid deletions eliminate polymerase activity; gray, segment in which deletions partially affect activity; white, segment in which deletions have minimal effect on activity; cyan, amino- and carboxy-terminal segments. Naturally occurring prolines at H_N _and H_C _are shown white-on-black.

Testing the contributions to RNA polymerase function of these two proposed actions of the bridge helix (which are not mutually exclusive) - or revealing other bridge-helix roles - is made difficult by the small movements involved relative to the size of the polymerase and by the inability of crystal structures to report molecular dynamics. Over the past few years, Weinzierl and colleagues have developed and exploited a novel approach that augments conventional structure-function studies by assaying RNA polymerase with systematically altered bridge-helix structures [6]. This ambitious undertaking was accomplished using robotics to assemble and assay many variants of an archaeal RNA polymerase (from *Methanocaldococcus jannaschii*) that can be reconstituted *in vitro *from individual subunits. The most recent results from this systematic dissection of the bridge helix, published in *BMC Biology *by Weinzierl [7], suggest that kinking of the helix at or adjacent to segments that contact the interconnected network of RNA polymerase modules may play a more important role in the nucleotide-addition cycle than contacting the template base or looping to generate a translocation pawl.

## Surprises in the conformational flexibility of the bridge helix

Significant conformational flexibility throughout the bridge helix is indicated by the presence of helix-destabilizing glycine residues at three to four conserved locations (Figure 1b). To detect regions in which the helix may be transiently disrupted during the nucleotide-addition cycle, Weinzierl [7] systematically substituted proline at every position in the helix. Most proline substitutions dramatically decreased total RNA synthesis on nicked calf-thymus DNA (the assay used in the robotic method). However, two substitutions, at positions 808 and 824 directly adjacent to conserved glycine residues (Cα spheres in Figure 1b), had the opposite effect of actually increasing total RNA synthesis. Interestingly, these positions correspond to the locations of naturally occurring prolines in the bridge helices of some bacterial RNA polymerases (for example, from *Bacillus subtilis*) or the newly described plant RNA polymerases IV and V (for example, from *Arabidopsis thaliana*). Thus, kinking focused at these two points of the bridge helix (Cα spheres in Figure 1b) appears not just to be tolerated, but to be stimulatory for RNA synthesis when facilitated by the presence of a proline residue.

Extension of these findings to investigate the curious presence of a deletion of two amino acids in the bridge helix of plant RNA polymerase IV (corresponding to the looped-out region proposed to act as a translocation pawl and shown as Cα-Cβ sticks in Figure 1b) led the author to a remarkable discovery that calls into question the translocation-pawl model. He reasoned that this deletion would radically twist the helix backbone and disrupt any coordinated looping-unlooping oscillations in the shortened region. Interestingly, the archaeal bridge helix tolerated a similar deletion without significant loss of activity not only at the polymerase IV location but also throughout the central portion of the bridge helix (white in Figure 1b). Lesser, but still significant, activity was observed in deletions near the amino-terminal proline substitution (gray in Figure 1b), whereas complete loss of activity was observed in deletions just amino- or carboxy-terminal to the proline substitutions (blue in Figure 1b). Bridge-helix regions that tolerated two-amino-acid deletions also tolerated proline substitution with only partial loss of activity. These results led Weinzierl to conclude that the proposed pawl-like function of the bridge helix or other proposed roles of this segment of the helix, such as contacting the template base, require re-evaluation because they are either redundant or do not exist for the archaeal helix.

## The bridge helix as a coordinator of conformational changes in RNA polymerase

**Figure 2:**
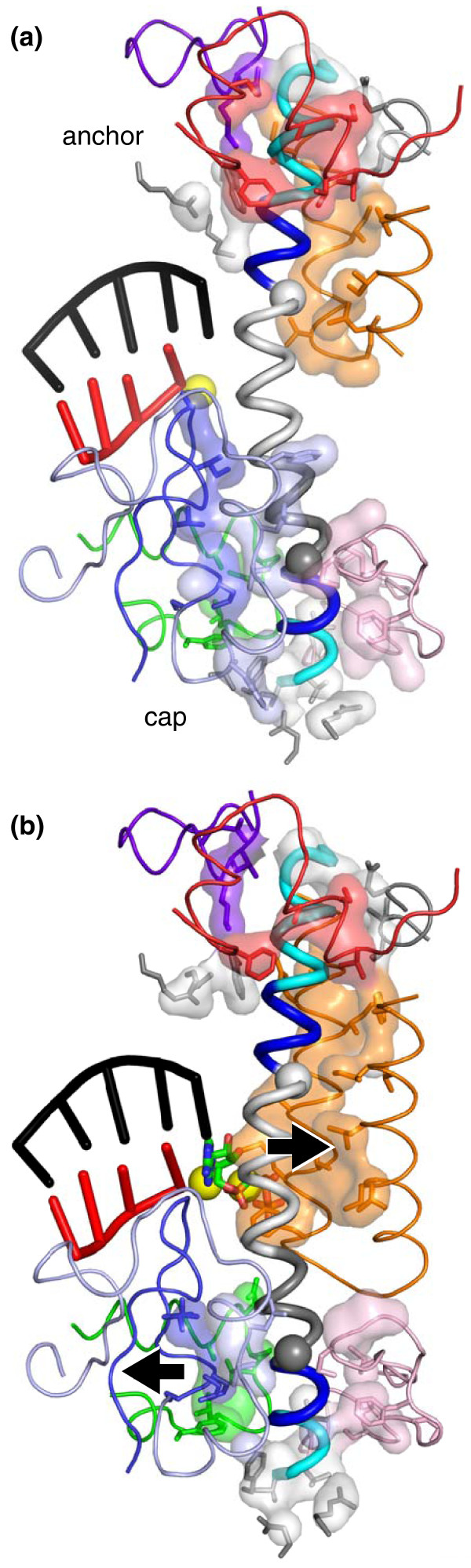
**RNA polymerase residues that contact the bridge helix**. DNA downstream of the active site is omitted for clarity. **(a) **Contacts in a *T. thermophilus *elongation complex lacking NTP (PDB 2o5i). **(b) **Contacts in a *T. thermophilus *elongation complex bound by α,β-methylene-ATP (PDB 2o5j). Residues that lie within 4 Å of the bridge helix (contacts) are shown as a semi-transparent surface and as sticks. Contacts occur principally in two regions, a cap that contacts the amino-terminal portion of the bridge helix and, in the NTP-bound complex, the trigger helices. Contacts made by polymerase loops or modules that change upon bridge-helix movements are color-coded: blue, RPB2/β D-loop; light blue, RPB2/β fork loop; green, RPB2/β link loop and helix; light pink, RPB1/β' F-loop; red, RPB1/β' switch 1; purple, RPB1/β' switch 5 and 11 adjacent residues; orange, RPB1/β' trigger loop or trigger helices. A portion of the trigger loop in the NTP-free elongation complex that does not contact the bridge helix is not shown and was not ordered in the structure. Other segments or individual side chains contacting the bridge helix are shown but not colored. Arrows indicate small movements of the bridge helix, D-loop, and fork loop (all approximately 1.5 Å) that occur upon substrate binding coupled to a larger movement of the RPB2/β lobe [3].

Together, Weinzierl's findings point instead to critical roles of bridge-helix segments that contact flexible loops in the polymerase on either side of the active site, the downstream DNA channel, and the secondary channel, through which NTPs enter the active site. He designates these segments as amino- and carboxy-terminal hinges (H_N _and H_C_), on the basis of the effects of the proline substitutions that increase polymerase activity (Cα spheres in Figures 1b and 2). H_N _and H_C _are adjacent both to highly conserved glycines that are likely to facilitate bridge-helix distortions and to regions that do not tolerate alteration (blue in Figures 1b and 2). Like Pro-Gly sequences that occur at the hinge points of the trigger loop-trigger helix transition, these hinge regions may facilitate helix distortions important to RNA polymerase function. Recently, Seibold *et al. *[8] also proposed that helix bending at H_N _facilitates catalysis.

The H_N_-proximal bridge-helix segment contacts four conserved loops in the polymerase that form a cap to the helix and that, in turn, make critical contacts to: the trigger helices (β'/RPB1 F-loop; light pink in Figure 2) [9]; the downstream fork junction of duplex and melted DNA (β/RPB2 fork loop; light blue in Figure 2) [3,8]; the NTP substrate (β/RPB2 D-loop; blue in Figure 2) [3]; and the nascent RNA, especially backtracked RNA (a β/RPB2 helix and loop termed the 'link domain' by Weinzierl [7]; green in Figure 2) [3,10]. The H_C_-proximal bridge-helix segment contacts the clamp domain and switch regions 1 and 5 (red and purple in Figure 2) in an anchor that changes conformation when the clamp changes position or upon formation of the trigger helices (Figure 2). When the trigger helices form, contacts of the bridge helix to the cap are reduced, consistent with movement of the central portion of the helix toward the trigger helices by 1.5 Å (Figure 2) [3]. Although this movement is modest, larger movements of the bridge helix occur in a wedged intermediate generated by α-amanitin binding to RNA polymerase II [11]. Facilitating these bridge-helix movements by increasing flexibility may explain the superactivity of proline substitutions at H_N _and H_C_. Furthermore, it is likely that these regions undergo other, and quite possibly larger, changes during steps of the nucleotide-addition cycle, including translocation, that remain to be captured by crystal structures. Thus, kinking of the bridge helix at H_N _and H_C _may allow it to coordinate conformational coupling between the two sides of the polymerase cleft in ways that remain to be elucidated.

In this view, bridge-helix conformation influences formation of the trigger helices (and thus catalysis) in response to DNA and RNA sequence or transcription regulators that interact with the RNA polymerase clamp, cap, or anchor and affect bridge-helix conformation through H_N _and H_C_. Loops observed in the central portion of the helix may be a simple consequence of its inherent instability as a helix, which optimally poises it to modulate trigger-helix formation, rather than making loop-specific contacts (for example, as a ratchet pawl). Such a view is consistent with impairment of catalysis by substitutions that disrupt fork loop-H_N _interaction [8] and with the general tolerance of the region between H_N _and H_C _to significant alterations such as the two-amino-acid deletions [7] and helix-destabilizing substitutions [5], as the effects on mediating regulatory signals may not be evident in a nonspecific transcription assay. It would also explain how the divergent bridge-helix sequences found in the plant RNA polymerases IV and V could accommodate robust DNA-dependent RNA synthesis. It bears emphasizing, however, that roles of the bridge helix in controlling catalysis via effects on formation of the trigger helices and in facilitating translocation are not mutually exclusive.

## Future studies of bridge-helix function

The results of Weinzierl's *tour de force *mutagenesis of the bridge helix [7] yield several important ideas about its function. Careful testing of predictions based on these ideas is now necessary to advance understanding of RNA polymerase structure and function. These predictions include: that significant conformational changes can occur in the amino-terminal portion of the bridge helix; that bridge-helix movements mediate changes in RNA polymerase activity via modules that interact with the amino- and carboxy-terminal portions of the bridge helix; and that the bridge-helix looping originally observed in DNA-free RNA polymerase structures plays no vital role in translocation. These tests will require examination of the bridge-helix variants described by Weinzierl [7] using biochemical assays that detect individual steps in the nucleotide-addition cycle, or of homologous alterations in other RNA polymerases for which a wider range of *in vitro *assays specific for individual steps in the cycle is available. The nonspecific RNA-synthesis assay used in the robotic approach does not identify which step in the cycle is stimulated by H_N _and H_C _proline substitutions or inhibited by other alterations; even template engagement could be affected, as faster recycling of RNA polymerase could also increase RNA yield. Thus, much important biochemistry remains before we will fully understand RNA polymerases. Among the most important objectives should be to devise an assay that unambiguously and directly reports effects on translocation. A second key goal should be the determination of additional crystal structures of DNA-bound RNA polymerases that capture more extensive conformational changes, such as clamp opening, that might reveal the predicted changes in bridge-helix conformation.
